# ﻿Molecular species delimitation and description of a new species of *Phenacogaster* (Teleostei, Characidae) from the southern Amazon basin

**DOI:** 10.3897/zookeys.1164.102436

**Published:** 2023-05-26

**Authors:** Camila S. Souza, George M. T. Mattox, George Vita, Luz E. Ochoa, Bruno F. Melo, Claudio Oliveira

**Affiliations:** 1 Departamento de Biologia Estrutural e Funcional, Instituto de Biociências, Universidade Estadual Paulista, R. Prof. Dr. Antônio C. W. Zanin 250, 18618-689, Botucatu, SP, Brazil Universidade Estadual Paulista Botucatu Brazil; 2 Laboratório de Ictiologia de Sorocaba, Departamento de Biologia, Universidade Federal de São Carlos, Rod. João Leme dos Santos km 110, 18052-780, Sorocaba, SP, Brazil Universidade Federal de São Carlos Sorocaba Brazil; 3 Museu de Zoologia da Universidade de São Paulo, Av. Nazaré 481, 04218-970, São Paulo, SP, Brazil Museu de Zoologia da Universidade de São Paulo São Paulo Brazil; 4 Dirección Académica, Universidad Nacional de Colombia, Sede de La Paz, Km 9 Via Valledupar, Los Robles La Paz, Cesar, Colombia Universidad Nacional de Colombia Los Robles La Paz Colombia; 5 Department of Ichthyology, American Museum of Natural History, 200 Central Park West, New York, NY 10024, USA American Museum of Natural History New York United States of America

**Keywords:** Biodiversity, Characinae, mitochondrial DNA, Neotropical freshwater fishes, Phenacogasterini

## Abstract

*Phenacogaster* is the most species-rich genus of the subfamily Characinae with 23 valid species broadly distributed in riverine systems of South America. Despite the taxonomic diversity of the genus, little has been advanced about its molecular diversity. A recent molecular phylogeny indicated the presence of undescribed species within *Phenacogaster* that is formally described here. We sampled 73 specimens of *Phenacogaster* and sequenced the mitochondrial cytochrome c oxidase subunit I (COI) gene in order to undertake species delimitation analyses and evaluate their intra- and interspecific genetic diversity. The results show the presence of 14 species, 13 of which are valid and one undescribed. The new species is known from the tributaries of the Xingu basin, the Rio das Mortes of the Araguaia basin, and the Rio Teles Pires of the Tapajós basin. It is distinguished by the incomplete lateral line, position of the humeral blotch near the pseudotympanum, and shape of the caudal-peduncle blotch. Meristic data and genetic differentiation relative to other *Phenacogaster* species represent strong evidence for the recognition of the new species and highlight the occurrence of an additional lineage of *P.franciscoensis*.

## ﻿Introduction

The Neotropical fish subfamily Characinae encompasses small- to medium-sized tetras found across South America and in Panama and Costa Rica (Lucena and Menezes 2003; Mattox et al. 2018). Most members of this subfamily have the anterodorsal region of the body with a gibbosity (except for *Acestrocephalus* Eigenmann, 1910 and *Phenacogaster* Eigenmann, 1907) and diverse trophic strategies, including carnivory, omnivory, and lepidophagy (Géry 1977; Sazima 1984). The subfamily sensu Souza et al. (2022) currently comprises 85 valid species distributed among seven genera: *Acanthocharax* Eigenmann, 1912, *Acestrocephalus*, *Charax* Scopoli, 1777, *Cynopotamus* Valenciennes, 1850, *Galeocharax* Fowler, 1910, *Phenacogaster*, and *Roeboides* Günther, 1864. *Phenacogaster* stands out as the largest and most taxonomically complex genus within Characinae, with 23 species distributed across cis-Andean South American riverine habitats (Fricke et al. 2023). They are small fishes measuring up to 6 cm standard length (SL) and are often known as “lambaris”, “glass tetras”, “mojaritas”, and “yaya” (Lucena and Malabarba 2010).

Relative to other Characinae genera, *Phenacogaster* possesses two longitudinal series of elongate and imbricated scales producing a zigzag pattern in a flat preventral region, as well as the outer premaxillary tooth row divided into a medial and a lateral section separated by a diastema (Eigenmann 1917; Malabarba and Lucena 1995; Mattox and Toledo-Piza 2012). Lucena and Malabarba (2010) presented the most comprehensive taxonomic revision of the genus with descriptions of nine species of *Phenacogaster*, nearly doubling the species diversity, and an identification key for the species, with the exception of the so-called *Phenacogasterpectinata* complex with *P.pectinata* (Cope, 1870), *P.microstictus* Eigenmann, 1909, *P.beni* Eigenmann, 1911 and *P.suborbitalis* (Ahl, 1936). Recently, three more species from the Brazilian Shield have been described: *P.naevata* Antonetti, Lucena & Lucena, 2018; *P.eurytaenia* Antonetti, Lucena & Lucena, 2018 from the Tocantins basin (Antonetti et al. 2018); and *P.julliae* Lucena & Lucena, 2019 from the Rio São Francisco (Lucena and Lucena 2019).

No study has been conducted to assess the interspecific genetic diversity of *Phenacogaster*, although species delimitation methods have been used for such purposes in other Characidae (Rossini et al. 2016; García-Melo et al. 2019; Brito et al. 2021; Malabarba et al. 2021; Mattox et al. 2023). A recent molecular phylogeny of Characinae revealed the presence of the two clades in *Phenacogaster*, the *P.pectinata* clade and the *P.franciscoensis* clade, as well as an undescribed species of *Phenacogaster* from the Xingu basin (Souza et al. 2022). To further investigate this question, we used mitochondrial data and species delimitation techniques to estimate intra- and interspecific genetic diversity within the genus. The results confirmed the presence of a new species in the upper Rio Xingu of the Amazonian Brazilian Shield, which is formally described in this paper.

## ﻿Materials and methods

### ﻿Taxon sampling

The molecular analysis encompassed 74 taxa (Suppl. material 4), including 73 specimens of *Phenacogaster* and *Tetragonopteruscarvalhoi* Melo, Benine, Mariguela & Oliveira, 2011 as an outgroup. Seventy sequences were generated, and four were retrieved from BOLD/GenBank: one *Tetragonopteruscarvalhoi*, two *P.wayana*, and one *P.calverti* (Suppl. material 4). We used *Phenacogaster* specimens collected or received from ichthyological collections, which were identified morphologically using identification key (Lucena and Malabarba 2010). All fishes were collected in accordance with Brazilian law through SISBIO/MMA permit no. 3,245, and collection, maintenance, and analyses procedures were conducted in accordance with international guidelines for animal experiments via CEEAA IBB/UNESP protocol no. 304.

### ﻿DNA amplification and sequencing

DNA was extracted from muscles or gills using the extraction method of Ivanova et al. (2006). The cytochrome c oxidase subunit I (COI) gene was amplified by polymerase chain reaction (PCR) using the FishF1/FishR1 and FishF2/FishR2 primers (Ward et al. 2005) or the L6252-Asn/H7271-COXI primers (Melo et al. 2011). PCR amplifications were performed in a total volume of 12.5 µl that included 1.25 µl of 10X buffer, 0.25 μl of MgCl_2_ (50 mM), 0.2 μl dNTPs (2 mM), 0.5 μl of each primer (5 mM), 0.1 μl of PHT Taq DNA polymerase (*Phoneutria*), 1.0 μl of genomic DNA (200 ng) and 8.7 μl ddH_2_O. The PCR conditions included an initial denaturation (5 min at 94 °C), 30 cycles of chain denaturation (50 s at 94 °C), primer hybridization (45 s at 50–54 °C), and nucleotide extension (1 min at 68°C), and a ﬁnal extension (10 min at 68 °C). All PCR products were checked on 1% agarose gels and then purified using ExoSap-IT (USB Corporation) according to the manufacturer’s instructions. The purified PCR products were subjected to sequencing procedures with the BigDye Terminator v. 3.1 Cycle Sequencing Ready Reaction Kit (Applied Biosystems) and purified by ethanol precipitation. PCR products were loaded onto an ABI 3130 DNA Analyzer automatic sequencer (Applied Biosystems).

### ﻿Molecular data analysis

Forward and reverse sequences were assembled using Geneious v. 7.1.9 (Kearse et al. 2012) and contigs aligned with MUSCLE (Edgar 2004) using the default parameters. Substitution saturation was determined using Xia et al. (2003)’s approach in DAMBE v5.3.38 (Xia 2013). Nucleotide variation, substitution patterns, and the best-fit model of nucleotide evolution were estimated in MEGA v. 10 (Kumar et al. 2018).

The maximum likelihood (ML) analysis was conducted using RAxML HPC-PTHREADS-SSE3 (Stamatakis 2014) with five random parsimony trees and the GTRGAMMA model on the *Zungaro* server at LBP/UNESP. The neighbor-joining (NJ) tree was estimated with the K2P+G model (Kimura 1980) and 1,000 bootstrap replicates in MEGA v10 (Kumar et al. 2018). Two species delimitation methods were performed: the Assemble Species by Automatic Partitioning (ASAP) analysis (Puillandre et al. 2021) via the webserver (https://bioinfo.mnhn.fr/abi/public/asap/asapweb.html) with Kimura (K80; 2.0); and the Poisson Tree Process (PTP; Zhang et al. 2013) using the ML tree as input, 100,000 generations, and other parameters at default in the PTP webserver (http://species.h-its.org/ptp/). MEGA v. 10 computed K2P+G distances across groups based on their morphological identification. The ASAP, PTP, and genetic distance analyses were conducted without the outgroup.

### ﻿Morphological analysis

Morphometric and meristic data were collected on the left side of every specimen whenever possible, following Malabarba and Lucena (1995) and Lucena and Gama (2007). Point-to-point measurements were taken with a precision of 0.1 mm using a digital caliper. Counts of vertebrae, supraneurals, gill rakers, and teeth were obtained from cleared and stained (c&s) specimens prepared in accordance with Taylor and Van Dyke’s (1985) methodology. Vertebral counts include the four centra of the Weberian apparatus as separate elements and the compound ural centrum as a single vertebra. Except for head subunits, which are reported as a percentage of head length (**HL**), other measurements are expressed as a percentage of standard length (**SL**). In the description, the frequency of each count is mentioned in parenthesis, and the holotype count is indicated with an asterisk. Institutional acronyms follow Sabaj (2020). Specimens from the Xingu basin were determined as types and specimens from Araguaia and Tapajós are listed as non-types. Examined material is organized by acronym and collection number, number of specimens, range of SL, locality, collection date, and collectors. Comparative material is classified according to the alphabetical order of species, and, within a species, it follows the same order as examined material.

## ﻿Results

### ﻿Molecular species delimitation

**Figure 1.**
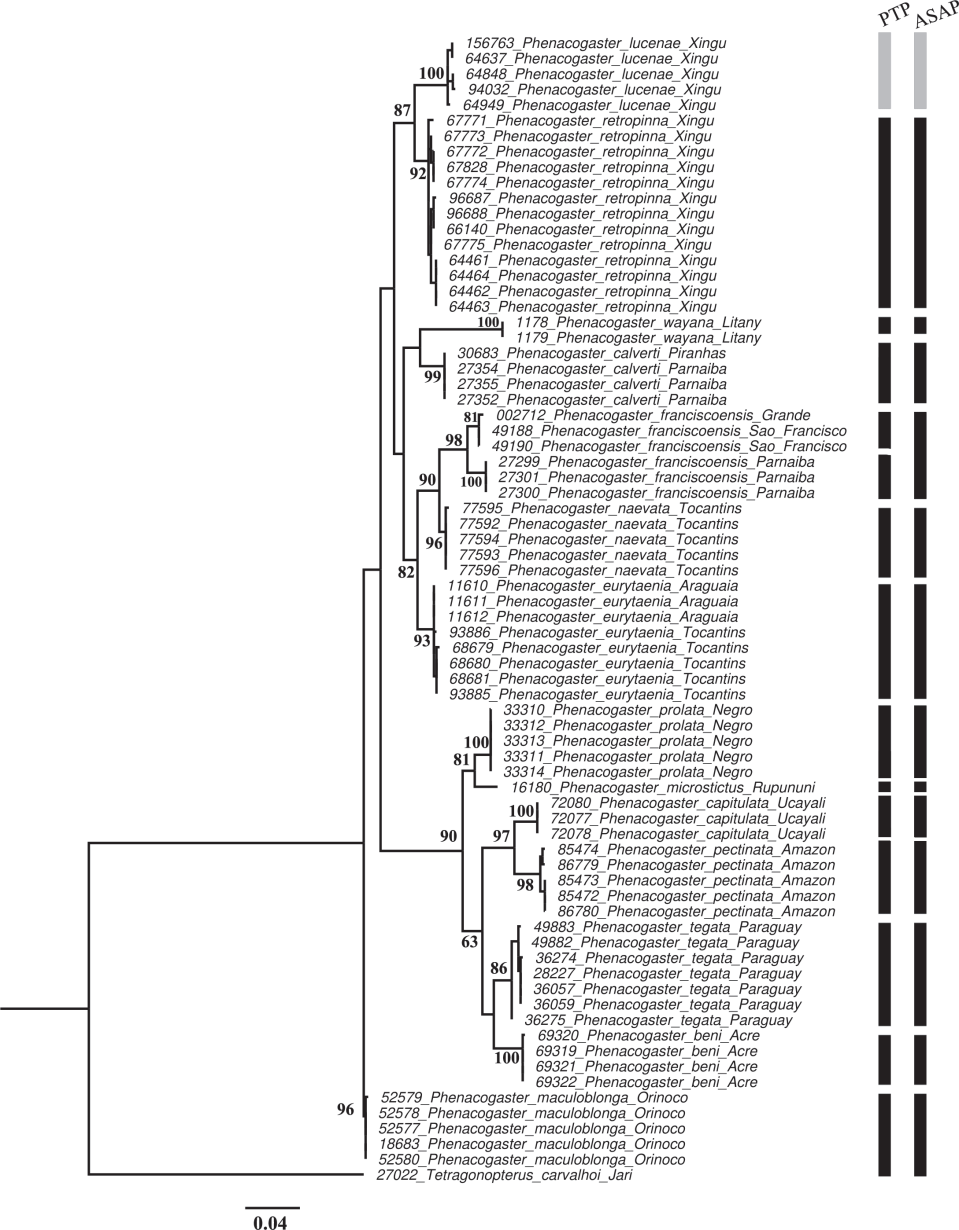
Maximum likelihood tree based on the cytochrome oxidase c subunit 1 gene (678 bp) of *Phenacogaster*. Vertical bars represent the number of species delimited by ASAP (14) and PTP (15). Gray bars represent the new species. Black bars indicate the other examined *Phenacogaster* species. Numbers near nodes represent bootstrap support for relevant nodes; values < 50% are not shown. Codes before tip names are tissue or database accession numbers.

Partial COI gene sequences were obtained from 68 specimens representing 13 of the 23 valid species of *Phenacogaster* (56.2%), and for five specimens that represent the species described in this study. The matrix consisted of 678 bp (153 variable sites) and had a nucleotide composition of 24.6% adenine, 27.5% cytosine, 18% guanine, and 30% thymine. In both asymmetrical (Iss.cAsym) and symmetrical (Iss.cSym) topologies, neither transitions nor transversions were found to be saturated by DAMBE. Both ML and NJ trees recovered similar topologies and supported the recognition of *P.lucenae* as a new species (Fig. 1, Suppl. material 1). The best partition provided by ASAP identified 14 species (1.00 asap-score) (Fig. 1, Suppl. material 2) and supported *P.lucenae* as new species. The PTP analysis defined 15 species and recognized the new species as a distinct lineage (Fig. 1, Suppl. material 3). Both methods recovered the same species limits, except for *P.franciscoensis* which was split in two by the PTP method. The overall mean K2P genetic distance was 0.077 ± 0.007. Interspecific genetic distances were between 0.026 ± 0.007 and 0.143 ± 0.020, and intraspecific genetic distances ranged between 0.000 ± 0.000 and 0.010 ± 0.003 (Table 1).

**Table 1.** Pairwise K2P genetic distances and intraspecific genetic variation of *Phenacogaster* species included in this study. Numbers below the diagonal represent the interspecific distance, while the numbers above the diagonal represent the relative standard deviation.

	1	2	3	4	5	6	7	8	9	10	11	12	13	14	Intraspecific genetic distances
1. *P.microstictus*		0.007	0.011	0.012	0.011	0.011	0.016	0.012	0.015	0.016	0.014	0.016	0.016	0.016	–
2. *P.prolata*	0.026		0.009	0.011	0.009	0.010	0.017	0.013	0.015	0.017	0.013	0.016	0.017	0.016	0
3. *P.capitulata*	0.050	0.042		0.008	0.010	0.011	0.021	0.015	0.018	0.019	0.017	0.017	0.022	0.020	0
4. *P.pectinata*	0.076	0.066	0.028		0.010	0.011	0.017	0.016	0.017	0.017	0.016	0.019	0.020	0.018	0.004±0.001
5. *P.beni*	0.058	0.052	0.045	0.061		0.008	0.016	0.014	0.016	0.017	0.014	0.016	0.018	0.018	0
6. *P.tegata*	0.058	0.053	0.046	0.069	0.039		0.016	0.013	0.015	0.015	0.014	0.017	0.016	0.017	0.003±0.001
7. *P.wayana*	0.102	0.111	0.123	0.125	0.111	0.110		0.014	0.012	0.016	0.013	0.017	0.014	0.016	0
8. *P.maculoblonga*	0.071	0.079	0.091	0.113	0.090	0.081	0.086		0.010	0.011	0.009	0.010	0.010	0.010	0.007±0.006
9. *P.calverti*	0.102	0.106	0.106	0.123	0.108	0.101	0.067	0.052		0.011	0.008	0.012	0.011	0.012	0
10. *P.franciscoensis*	0.114	0.118	0.123	0.131	0.125	0.114	0.106	0.068	0.069		0.010	0.008	0.012	0.012	0.010±0.003
11. *P.eurytaenia*	0.092	0.088	0.103	0.118	0.097	0.093	0.073	0.049	0.042	0.051		0.008	0.010	0.012	0.001±0.001
12. *P.naevata*	0.094	0.094	0.107	0.120	0.100	0.098	0.085	0.050	0.063	0.034	0.029		0.013	0.015	0
13. *P.retropinna*	0.110	0.110	0.129	0.143	0.126	0.110	0.080	0.053	0.058	0.073	0.050	0.058		0.008	0.005±0.002
14. *P.lucenae*	0.107	0.104	0.116	0.131	0.123	0.111	0.100	0.051	0.067	0.076	0.061	0.067	0.038		0.003±0.001

### ﻿Taxonomy

#### ﻿ Phenacogaster lucenae sp. nov.

Taxon classificationAnimaliaCharaciformesCharacidae

6E0DB71B-9823-5787-B608-74ECE1FB566F

https://zoobank.org/22F1F5CC-705D-406D-BA0F-7386E434C963

Fig. 2 , Table 2

Phenacogaster sp. Xingu: Souza et al. 2022: 9, figs 3, 5 [molecular phylogeny; cited in figures also as Phenacogaster sp. Xingu].

##### Type material.

***Holotype*.** MZUSP 126754, 26.7 mm SL, Brazil, Pará, Novo Progresso, Amazon basin, Rio Xingu, stream affluent of Rio Curuá, 08°29'59"S, 54°58'06.1"W, 08 Aug 2015, F.C.P Dagosta, M.M.F. Marinho, P. Camelier, V. Giovannetti.

***Paratypes***: All from Brazil, Amazon basin, Rio Xingu. LBP 15807, 2, 21.5–28.1 mm SL, Mato Grosso, Querência, Rio Feio, 12°33'20.5"S, 52°16'16.1"W, 31 Jul 2012, C. Oliveira, M. Taylor, G.J.C. Silva, J.H.M. Martinez. LBP 15835, 2, (tissue: 64949) 19.3–26.9 mm SL, Mato Grosso, Querência, Rio Suiá-Missu, Rio Feio, 12°31'55.7"S, 52°20'29.8"W, 31 Jul 2012, C. Oliveira, M. Taylor, G.J.C. Silva, J.H.M. Martinez. LBP 16061, 9, 22.9–35.4 mm SL, Mato Grosso, Primavera do Leste, Rio Culuene, Córrego Xavante, 14°38'24"S, 53°55'38"W, 05 Aug 2012, C. Oliveira, M. Taylor, G.J.C. Silva, J.H.M. Martinez. LBP 25217, 1, (tissue: 94032) 30.5 mm SL, Pará, Altamira, Rio Treze de Maio, 08°39'06.9"S, 55°02'09.1"W, 24 Sep 2017, A.C. Dias, C.S. Souza, C. Souto, N. Flausino Jr, R. Devidé. LBP 30738, 1, 38.0 mm SL, Mato Grosso, Primavera do Leste, Rio Culuene, Córrego Xavante, 14°38'24"S, 53°55'38"W, 23 Aug 2021, C.S. Souza, L. Reia, G.S.C. Silva, E.V. Ywamoto. LBP 32224, 1, 30.3 mm SL, Pará, Altamira, Castelo dos Sonhos, Rio Iriri, waterfall in Rio Curuá, 08°19'07"S, 55°05'22"W, 23 Aug 2022, T. Faria, G.S.C. Silva. MZUSP 97621, 49, 21.6–33.6 mm SL (7 c&s, 22.0–32.5 mm SL), Pará, Altamira, Amazon basin, Rio Curuá-Iriri, 08°15'17"S, 55°06'40"W, 27 Oct 2007, J.L.O. Birindelli, L.M. Souza, A.L. Netto-Ferreira, M.H. Sabaj, N.K. Lujan. MZUSP 120058, 28, 20.4–34.9 mm SL (5 c&s, 22.7–29.9 mm SL), collected with holotype.

##### Non-type specimens.

LBP 32258, 1, 28.3 mm SL, Mato Grosso, Sinop, Rio Tapajós, Rio Teles Pires, Arroio São José, Tujá, 11°36'04.47"S, 55°25'37.79"W, 25 Aug 2022, T. Faria, G.S.C. Silva. LBP 32321, 14, 19.0–25.7 mm SL (4 c&s, 18.8–22.7 mm SL), Mato Grosso, Guarantã do Norte, Rio Tapajós, Rio Teles Pires, igarapé of Rio Braço Norte, 09°56'54"S, 55°01'50"W, 24 Aug 2022, T. Faria, G.S.C. Silva. LBP 32332, 1, 22.6 mm SL, Mato Grosso, Sinop, Rio Tapajós, Rio Teles Pires, Arroio São José, Tujá, 11°36'04.47"S, 55°25'37.79"W, 27 Aug 2022, T. Faria, G.S.C. Silva. MZUSP 97708, 8, 27.3–32.4 mm SL, Mato Grosso, Santo Antonio do Leste, Araguaia basin, Rio das Mortes, Rio Suspiro, 14°52'30.0"S, 54°05'0.0"W, 18 Jan 2002, N.A. Menezes, O.T. Oyakawa, G.M. Guazzelli, R. Quevedo. MZUSP 118678, 8, 26.9–29.6, Mato Grosso, Santo Antonio do Leste, Araguaia basin, Rio das Mortes, Rio Suspiro, 14°52'30.92"S, 54°05'1.47"W, 17 Nov 2014, F.C.P. Dagosta, W.M. Ohara, V. Giovannetti.

##### Diagnosis.

*Phenacogasterlucenae* is distinguished from all congeners except *P.tegata* (Eigenmann, 1911), *P.carteri* (Norman, 1934), *P.napoatilis* Lucena & Malabarba, 2010, and *P.capitulata* Lucena & Malabarba, 2010 by having an incomplete lateral line (vs. complete lateral line). It differs from *P.tegata* by the presence of a round or slightly longitudinal oval humeral blotch near the pseudotympanum and distant from the vertical through dorsal-fin origin (vs. humeral blotch longitudinally elongated distant from pseudotympanum, closer to vertical through dorsal-fin origin). The new species differs from *P.carteri* by having a humeral blotch in males and females (vs. absence of humeral blotch in both sexes) and from *P.napoatilis* and *P.capitulata* by having a humeral blotch in both sexes (vs. absence of humeral blotch in males). In addition to the incomplete lateral line (vs. complete), *P.lucenae* differs from *P.retropinna* Lucena & Malabarba, 2010 by the anal-fin origin at vertical through base of first or second dorsal-fin branched ray (vs. anal-fin origin located posteriorly to that point), and from *P.ojitata* Lucena & Malabarba, 2010 by the round caudal peduncle blotch slightly reaching over the middle caudal-fin rays (vs. a diamond-shaped caudal peduncle blotch and further extending over the middle caudal-fin rays).

##### Description.

Morphometric data summarized in Table 2. Body compressed. Dorsal profile convex from anterior tip of upper jaw to origin of dorsal fin with a slight concavity in occipital region; slightly straight from dorsal-fin base to origin of adipose fin and slightly concave from that point to base of dorsal procurrent caudal-fin rays. Ventral profile of body convex from tip of lower jaw to anal-fin origin, straight along anal-fin base, straight to slightly concave from that point to ventral procurrent caudal-fin rays. Preventral area flattened with two longitudinal series of elongate scales overlapping; scales different in shape from remaining body scales and forming zigzag pattern in ventral view. Pseudotympanum triangular extending from region of rib of fifth vertebra to anterior border of rib of sixth vertebra.

**Table 2.** Morphometric data of *Phenacogasterlucenae* (*n* = 32 including holotype and paratypes). All from the Rio Xingu. Range includes holotype. SD = standard deviation.

	Holotype	Range	Mean	SD
Standard length (SL) (mm)	26.7	24.1–38	29.5	–
**Percentages of standard length**				
Greatest body depth	31.5	29.4–36.2	32.6	1.8
Snout to dorsal-fin origin	53.3	50.6–55.3	53.1	1.1
Snout to pectoral-fin origin	26.7	26.6–31.5	28.5	1.3
Snout to pelvic-fin origin	42.7	39.1–44.	41.6	1.2
Snout to anal-fin origin	53.8	51.3–58.9	55.4	2.0
Dorsal-fin origin to hypural joint	51.5	48–54.3	51.2	1.6
Dorsal-fin origin to anal-fin origin	31.2	30.2–36.9	33.0	1.7
Dorsal-fin origin to pelvic-fin origin	32.3	31.9–37.6	34.4	1.5
Dorsal-fin origin to pectoral-fin origin	38.9	36–41.3	38.4	1.4
Caudal-peduncle depth	9.1	8.6–11.4	9.8	0.7
Pectoral-fin length	16.6	16.5–22.8	20.2	1.8
Pelvic-fin length	15.2	14.1–20.4	17.5	1.6
Head length	28	24–29.5	27.1	1.2
**Percentages of head length**				
Snout length	26.5	23.4–31.4	26.8	1.9
Orbital diameter	36.2	34–42.9	37.7	2.1
Interorbital width	27	24.4 – 31.5	27.5	1.9

Mouth terminal, lower jaw slightly shorter than upper jaw; posterior tip of maxilla reaching vertical at midpoint of second infraorbital. Premaxillary teeth in two rows. Outer row with 6(4), 7(3), 8(4), 9(4), or 10(1) total teeth, divided in medial and lateral sections by gap; medial section with 2(6), 3(9) or 4(1) tricuspid teeth; lateral section with 3(1), 4(4), 5(5), or 6(6) conical teeth. Inner row with 8(1), 9(2), 10(6), 11(4), or 12(3) teeth, 3(2), 4(6), or 5(8) tricuspid teeth followed by 4(3), 5(1), 6(7), 7(3), or 8(2) conical teeth. Maxilla with 20(1), 21(2), 22(1), 23(1), 24(1), 25(1), 26(2), 27(5), or 29(1) conical teeth. Dentary with single row of 14(1), 15(1), 16(1), 17(3), 18(5), 19 (3), 20(1), or 21(1) teeth, with 4(2), 5(1), 6(7), 7(5), or 8(1) tricuspid teeth followed by 10(3), 11(5), 12(5), 13(1), or 14(2) conical teeth (Fig. 3).

Dorsal-fin rays ii,8(7) or 9*(17). Anal-fin rays iii-v,29(2), 30(8), 31(3), 32*(4), 33(6), or 34(1). Pectoral fin rays i,11*(13) or i,12(12). Pelvic-fin rays i,7*(28); its tip reaching beyond anal-fin origin. Lateral line incomplete. Longitudinal line of scales 32(2), 33(2), 34*(19), or 36(4). Pored scales 8(6), 9*(9), 10(3), 11(5), 12(4), 13(1), 14 (3), or 16(1); some specimens with 2(3) or 3(3) perforated scales anterior to last vertical series of scales. Scale series between lateral line and dorsal-fin origin 5(3), 6*(22), or 7(5). Scale series between lateral line and anal-fin origin 4(12), 5*(14), or 6(4). Gill rakers on upper limb of first gill arch 3(10) or 4(6); gill rakers on lower limb 7(10) or 8(6). Total vertebrae 33(1), 35(9), 36(1), or 37(1): precaudal 14(1), 15(11), or 16(1), caudal 19(1), 20(9), or 21(3). Supraneurals 3(1), 4(13), or 5(2).

##### Color in alcohol.

**Figure 2.**
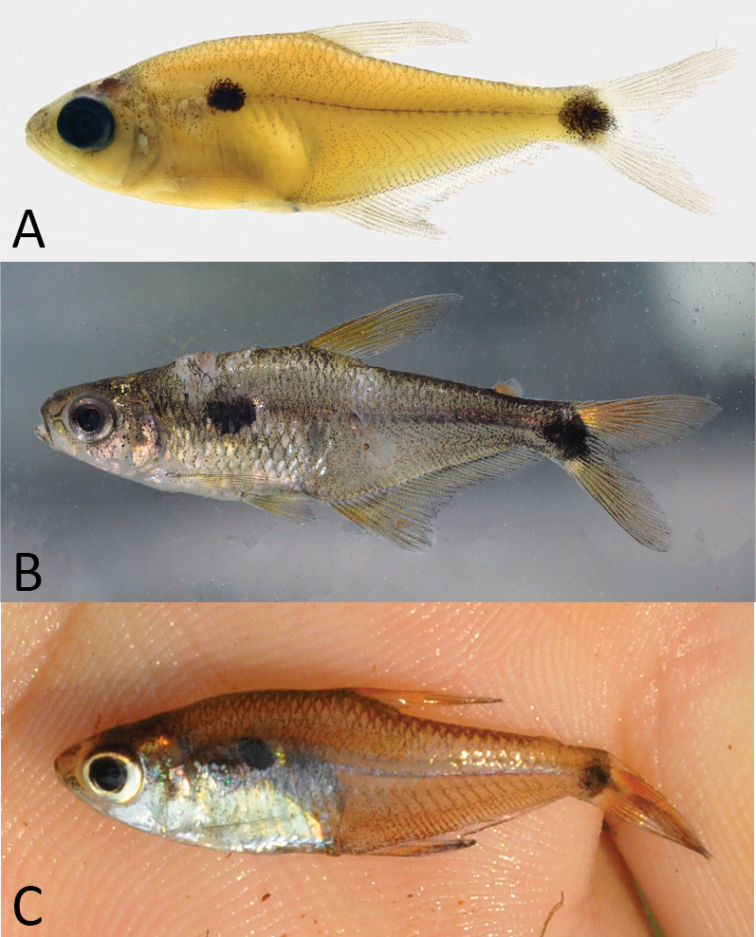
*Phenacogasterlucenae***A** MZUSP 126754, holotype, 26.7 mm SL, Brazil, Pará, Novo Progresso, Xingu basin, stream affluent of Rio Curuá **B** LBP 30738, paratype, 38.1 mm SL, Brazil, Mato Grosso, Primavera do Leste, Xingu basin, Rio Culuene, Córrego Xavante **C** LBP 25217, paratype, 30.6 mm SL, Brazil, Pará, Altamira, Xingu basin, Rio Treze de Maio.

Overall ground coloration pale yellow (Fig. 2A). Dorsolateral region of body with melanophores along margins of scales. Ventrolateral region less pigmented. Thin lines of melanophores accompanying myosepta along flanks, more evident in the hypaxial musculature. Females and males with rounded or slightly longitudinally oval humeral blotch immediately posterior to pseudotympanum, covering roughly three to five scale rows vertically and two to five scales longitudinally. Caudal peduncle with circular patch of melanophores covering whole caudal peduncle depth and reaching base of caudal-fin middle rays. Thin line of melanophores extending along horizontal septum between humeral and caudal peduncle blotches. Anal, pelvic, pectoral, and dorsal fins scattered by small melanophores. Adipose fin hyaline (Fig. 2).

**Figure 3.**
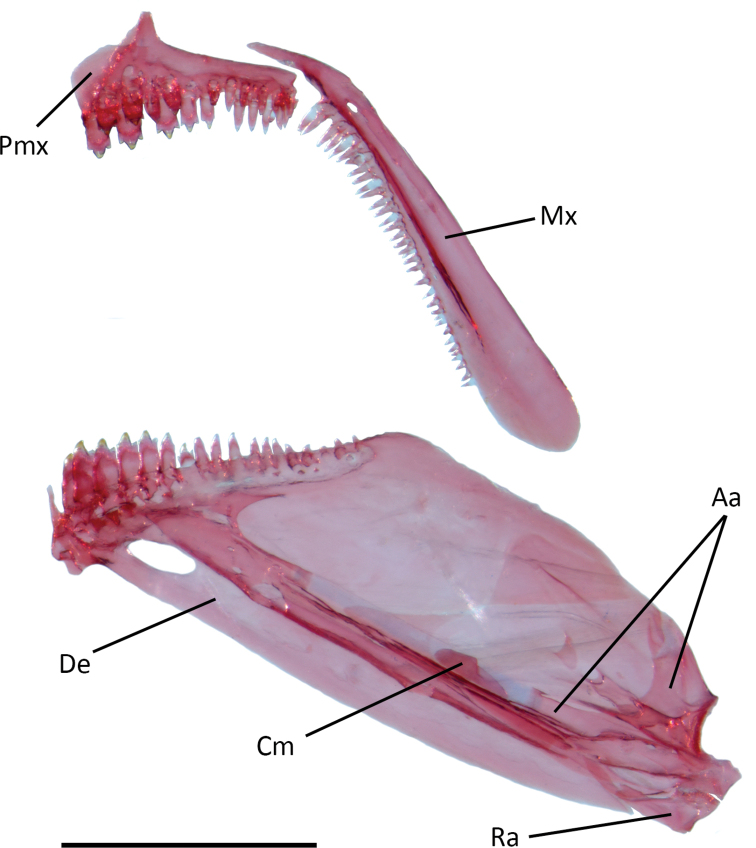
*Phenacogasterlucenae*, MZUSP 97621, 24.9 mm SL, c&s paratype, lateral view, right side, image flipped. Abbreviations: Aa, anguloarticular; Cm, coronomeckelian; De, dentary; Mx, maxilla; Pmx, premaxilla; Ra, retroarticular. Scale bar: 1 mm.

##### Color in life.

Overall ground coloration yellowish to golden on slightly translucent background (Fig. 2B, C). Dorsolateral body region with melanophores along margins of scales. Ventrolateral area less pigmented. Humeral blotch rounded or oval with anterior portion black and posterior edge iridescent yellow to orange. Round black blotch on middle portion of caudal peduncle extending vertically over entire caudal peduncle depth and extending posteriorly to proximal portion of caudal-fin middle rays. Some specimens with bright golden or white patches on posterior portion of caudal peduncle blotch, covering base of caudal-fin rays in upper and lower lobes. Thin line of melanophores between humeral and caudal peduncle blotches. Abdominal cavity, opercular series and portion of infraorbitals covered with guanine. All fins orange to yellowish coloration, with anterior halves of caudal-fin lobes more intensely colored. Posterior tip of caudal and dorsal fins scattered by small dark chromatophores (Fig. 2B, C).

##### Sexual dimorphism.

**Figure 4.**
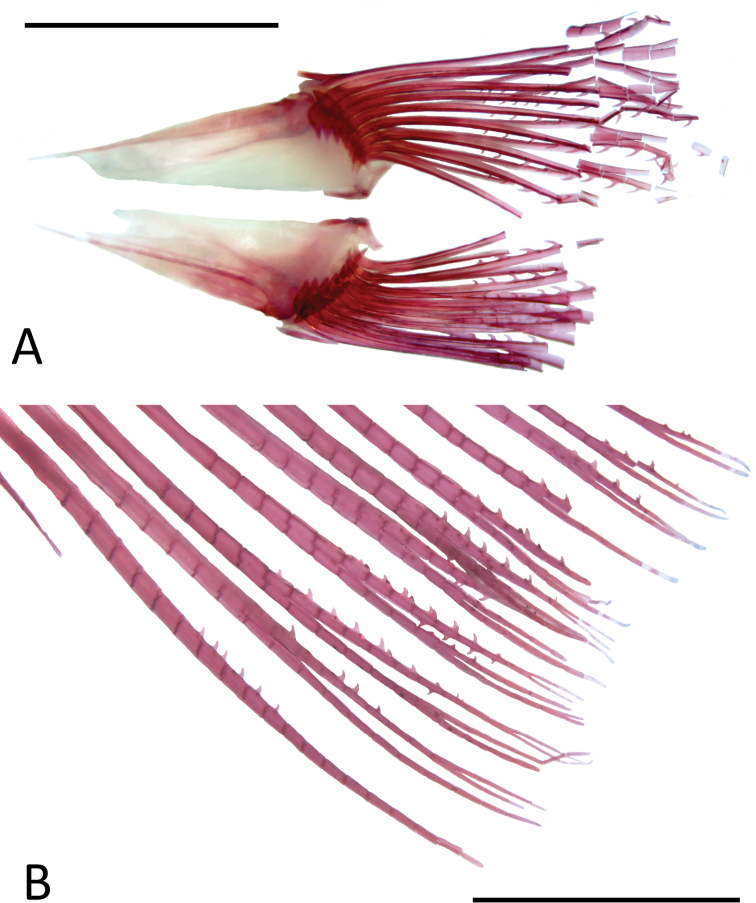
*Phenacogasterlucenae*, MZUSP 97621, 30.4 mm SL, c&s paratype, male **A** pelvic-girdle, ventral view, distal tip of rays damaged **B** anal-fin rays in lateral view. Scale bars: 2 mm.

Our samples consist of two adult males (MZUSP 97621, 30.4–34.4 mm SL) with hooks on pelvic- and anal-fin rays (Fig. 4). Four to six lateralmost branched pelvic-fin rays with five to nine curved hooks on medial edge of rays, one hook per segment towards the tips and more hooks per segment toward the base of the rays. Hooks more developed and frequent on medial regions of branched rays (Fig. 4A). Anal-fin rays with four to nine curved hooks on the posterior edges of the last unbranched and the first to eleventh branched fin rays. Fin hooks more developed and abundant on anterior branched rays. In most cases, one pair of small hooks per segment, but occasionally two pairs per segment. Hooks in some cases incipient in the form of bumps. A few rays with additional single hook on the anterior edge of distal portion (Fig. 4B).

##### Distribution and habitat.

**Figure 5.**
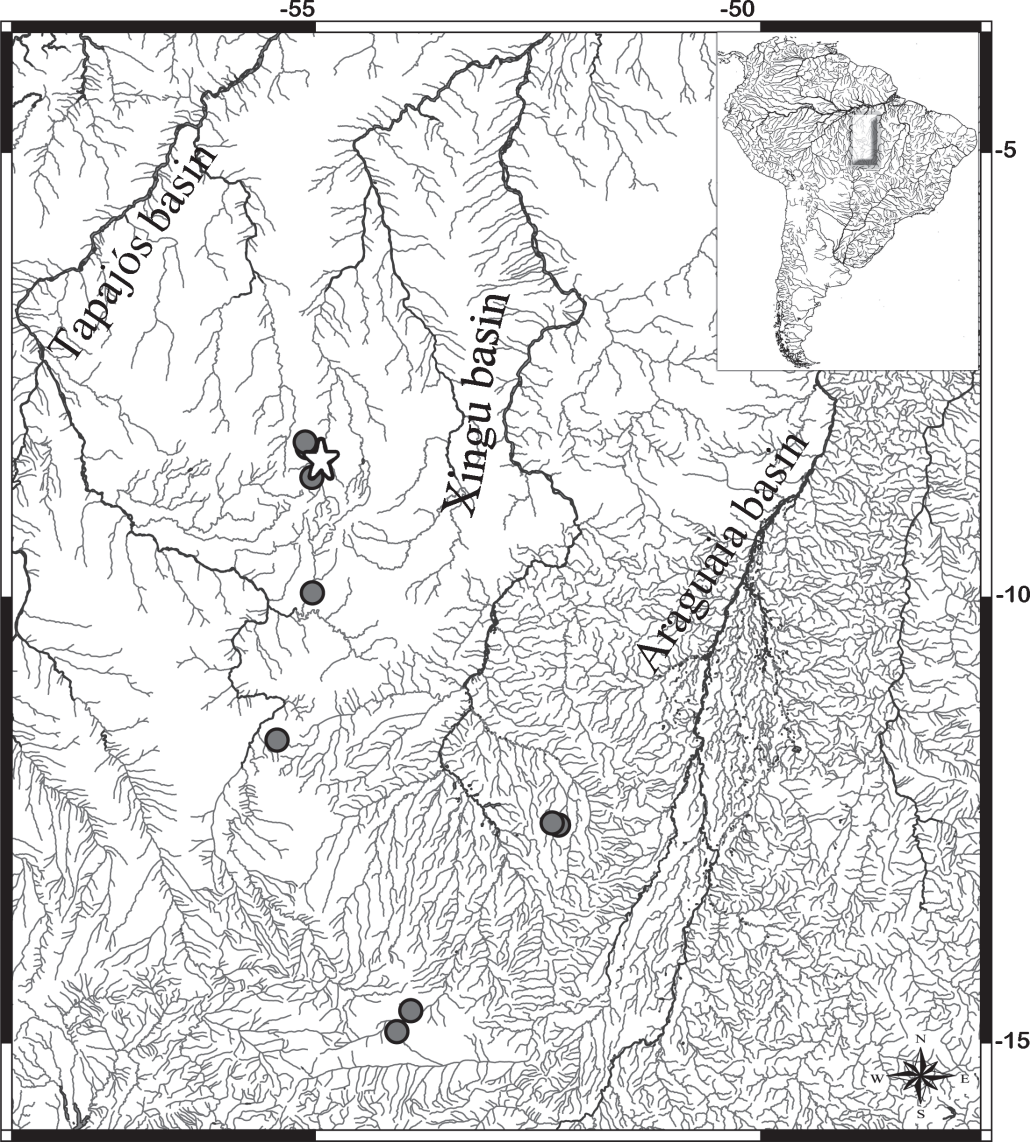
Map showing the distribution of *Phenacogasterlucenae* in the upper Xingu, Tapajós, and Araguaia basins. White star represents the type locality at tributary of Rio Curuá, Xingu basin.

**Figure 6.**
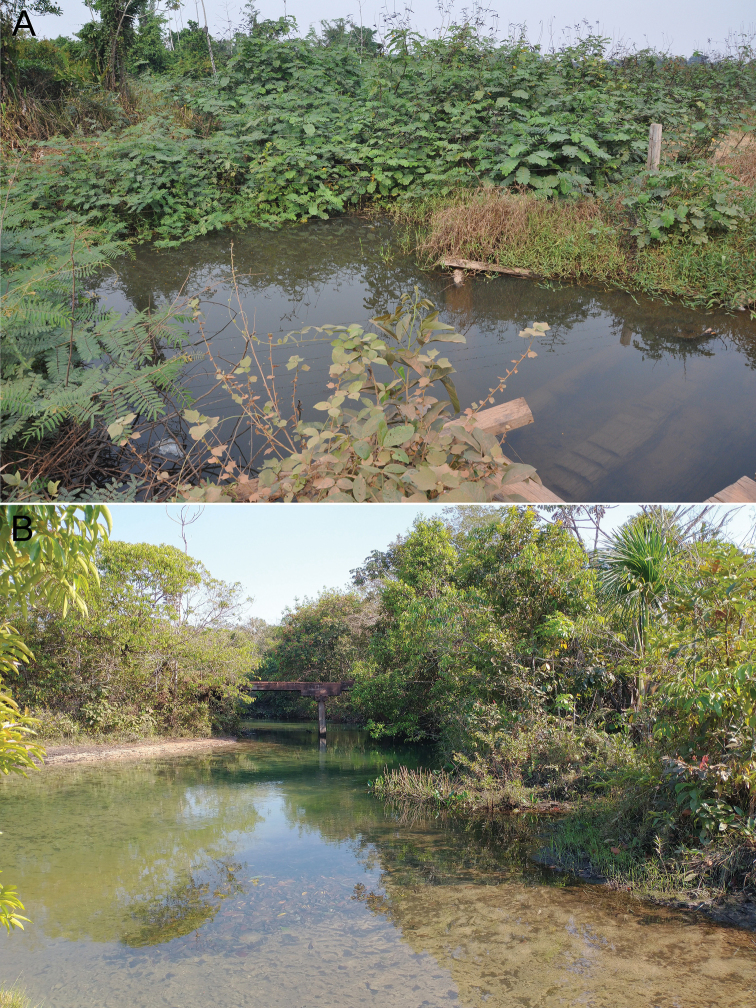
Habitats of *Phenacogasterlucenae***A** Rio Treze de Maio, Xingu basin, Altamira, Pará, Brazil, 08°39'06.9"S, 55°02'09.1"W (LBP 25217) **B** Córrego Xavante, Rio Culuene, Xingu basin, Mato Grosso, Primavera do Leste, Brazil, 14°38'24"S, 53°55'38"W (LBP 30738). Photographs by CS Souza.

*Phenacogasterlucenae* is known from tributaries of the Rio Curuá-Iriri, Rio Culuene, and Rio Suiá-Miçu (upper Xingu basin), tributaries of Rio das Mortes (upper Araguaia basin), and Rio Teles Pires (upper Tapajós basin), Amazon basin, Pará and Mato Grosso states, Brazil (Fig. 5). The new species was found in association with marginal vegetation (Fig. 6).

##### Etymology.

*Phenacogasterlucenae* is named in honor of Dr. Zilda Margarete Seixas de Lucena, an eminent ichthyologist who has significantly contributed to our knowledge of *Phenacogaster* taxonomy. A noun in genitive case.

##### Conservation status.

*Phenacogasterlucenae* is found in the upper Xingu, Araguaia, and Tapajós basins, where specimens were collected during focused expeditions. Although deforestation and hydroelectric plants have affected the region, 18 specimens of *P.lucenae* have been collected in recent years (2021–2022), demonstrating a likely high resilience to anthropogenic impacts. Therefore, we suggest the categorization Least Concern (LC) according to the International Union for Conservation of Nature criteria (IUCN 2014, Standards and Petitions Subcommittee).

##### Comparative material examined.

***Phenacogastercapitulata***: LBP 17802 (6, 27.38–31.97 mm SL), Peru, Pucallpa, Coronel Portillo, Ucayali basin, 08°35'44.2"S, 74°48'04.3"W, 18 Jun 2013, R. Britzke. ***P.napoatilis***: MZUSP 38667 (9, 21.5–35 mm SL), Equador, Napo, Napo basin, Rio Jatuncocha, 2km above Laguna Jatuncocha, 1°3'0.00"S, 75°31'4.0"W, 26 Oct 1981, D. Stewart & M. Ibarra. ***P.ojitata***: MZUSP 30551 (36.3 mm SL), Brazil, Pará, Rio Curuá, Serra do Cachimbo, rodovia Santarém- Cuiabá, poço de cachoeira, 09°22'0.0"S, 54°52'0.0"W, 15 Aug 1984, M Goulding. MZUSP 97588 (9, 30.5–48.8 mm SL), Brazil, Pará, Altamira, Xingu basin, Rio Curuá, na ponte da BR163, 08°53'54"S, 55°59'20"W, 29 Oct 2007, J. Birindelli, L. Sousa, A. Netto-Ferreira, M. Sabaj, N. Lujan. MZUSP 100922 (22, 28.4–33.5 mm SL; 2 d&c, 31.2–30.5 mm SL), Brazil, Pará, Rio Curuá, Serra do Cachimbo, rodovia Santarém-Cuiabá, poço de cachoeira. ***P.retropinna***: LBP 15676 (105, 21.5–43.3 mm SL), Brazil, Mato Grosso, Ribeirão Cascalheira, Xingu basin, Córrego do Gato, 13°09'13.6"S, 51°55'18.7"W, 30 Jul 2012, C. Oliveira, M. Taylor, G. Silva, J. Henriques. LBP 25926 (2, 33.6–36.4 mm SL), Brazil, Mato Grosso, Paranatinga, Xingu basin, Rio Culuene, 13°50'50.8"S, 53°15'40.2"W, 24 Jan 2018, N.F. Junior, N. Estevão, F.A. Machado. MZUSP 30550 (12, 18.5–30.5 mm SL), Brazil, Mato Grosso, Gaúcha do Norte, Rio Xingu, mouth of Rio Culuene and Sete de Setembro, 12°56'0.0"S, 52°49'0.0"W, 23 Jul 1984, M. Goulding, Portugal & Carvalho. MZUSP 81267 (17, 32.9–39.2 mm SL), Brazil, Amazonas, Rio Negro, 00°16'22.0"N, 69°54'3.0"W, 7 Nov 2002, F. Lima et al. MZUSP 99771 (14, 32.0– 40.3 mm SL), Brazil, Mato Grosso, Aripuanã, Madeira basin, Rio Aripuanã, Balneário Primavera, a jusante do salto de Dardanelos, 10°09'54"S, 59°26'55"W, 12 Dec 2004, F. Machado, C. Leite, N. Silva, R. Rosa. ***P.tegata***: LBP 7606 (16, 21.7–31.8 mm SL), Brazil, Mato Grosso, Barão de Melgaço, Paraguay basin, Lagoa Marginal rio Cuiabá, 16°11'39.5"S, 55°48'25.1"W, 29 Jan 2021, C. Oliveira, G.A. Lopez, R. Britzke. LBP 7641 (6, 35.8–39.1 mm SL), Brazil, Mato Grosso, Santo Antonio do Leverger, Paraguay basin, 15°46'03.8"S, 55°30'44.5"W, 01 Mar 2009, M. Mehanna, P.A. Campos. MZUSP 35889 (5, 26.9–37.9 mm SL), Brazil, Mato Grosso, Itiquira, Paraguay basin, Rio Piquiri, faz. Santo Antônio do Paraíso, 17°12'0.0"S, 54°9"0.0"W, J.H.B. Medeiros, J.C. Oliveira. MZUSP 96694 (10, 24.8–27.7 mm SL), Brazil, Mato Grosso, Barão do Melgaço, Paraguay basin, Rio Mutum.16°19'30"S, 55°49'59"W, F.A. Machado et al.

## ﻿Discussion

This is the first molecular delimitation using barcode sequences of the genus *Phenacogaster* spanning more than half of the known species diversity and supplements the phylogenetic study of the Characinae recently published including 16 species of *Phenacogaster* (Souza et al. 2022). Based on the application of the species delimitation methods, results identified 14 or 15 species (ASAP and PTP) and both supported *P.lucenae* as a new species (Fig. 1). The minor difference of ASAP and PTP results may be attributable to the range of algorithms and implementations, population size, species diversity, and speciation rates (Ahrens et al. 2016; Puillandre et al. 2021). The species delimitation methods are useful tools that, when combined with other types of information such as morphological data, may constitute solid evidence for species delimitation (Melo et al. 2016; Mateussi et al. 2020; Lozano et al. 2022).

The *Phenacogasterpectinata* complex (*P.pectinata*, *P.microstictus*, *P.suborbitalis* and *P.beni*) was proposed for widely distributed species characterized by humeral blotch present only in females, humeral blotch absent or restricted to a few chromatophores in males, complete lateral line, and 32–42 branched anal-fin rays (Lucena and Malabarba 2010). Phylogeny based on genomic evidence supports the group (*P.pectinata* clade) and adds *P.capitulata*, *P.megalostictus*, *P.prolata*, *P.suborbitalis*, and *P.tegata* (Souza et al. 2022). Both topologies of our study concur with the molecular phylogeny (Fig. 1, Suppl. material 1) and adds *P.microstictus* from the Rupununi River as another member of the clade closer to *P.prolata* from the Negro basin (Fig. 1).

*Phenacogasterlucenae* belongs to the *P.franciscoensis* clade (Souza et al. 2022). In fact, these authors sequenced ultraconserved elements for *P.lucenae* (identified there as *Phenacogaster* sp. Xingu) and discovered that it is the sister species of *P.retropinna* (Tapajós and Xingu) (Souza et al. 2022). Lucena and Malabarba (2010) described *P.retropinna* from the Amazonian rivers Negro, Madeira, Xingu, and Araguaia. Here, both molecular and morphological evidence support the distinction between *P.lucenae* and *P.retropinna*. The mitochondrial data analysis revealed a reasonably high genetic divergence (0.038±0.008) between these species (Table 1, Fig. 1).

Lucena and Malabarba (2010) described the endemic *Phenacogasterojitata* from the Rio Curuá, a left tributary of the Xingu. Unfortunately, there are currently no tissues of *P.ojitata* for molecular analyses. Morphologically, *P.lucenae* can be recognized from *P.ojitata* by the round caudal peduncle blotch slightly reaching over the middle caudal-fin rays and a larger orbital diameter (34–42.9% of HL; see diagnosis). In addition, *P.lucenae* can be distinguished from other *Phenacogaster* with incomplete lateral line by the presence of humeral blotch (vs. absence of humeral blotch in *P.carteri*), presence of humeral blotch in males and females (vs. absence of humeral blotch in males of *P.napoatilis* and *P.capitulata*); humeral blotch near pseudotympanum and distant from vertical line through dorsal-fin origin in males and females (vs. humeral blotch distant from pseudotympanum and near dorsal-fin origin in males and females of *P.tegata*).

Reduction or lack of pores in the laterosensory system is a classic reductive trait in fishes (Myers 1958) and most likely results from the loss of terminal developmental stages as consequence of the body size reduction (Marinho et al. 2021). As stated previously, the incomplete lateral line is present in four of the currently 23 valid species of *Phenacogaster* (Lucena and Malabarba 2010) in addition to *P.lucenae* described here. Although we did not have access to tissue samples from all these species, our results indicate that reduction of the lateral line independently evolved three times in the phylogeny (Souza et al. 2022). We detected incomplete lateral lines in both juveniles and adults of *P.lucenae*, with only six of 32 specimens exhibiting scale interruptions along the lateral line (i.e., incomplete pored series with two or three pored scales towards the end of the scale series, and a long gap of non-pored scales between them). The evolutionary significance for this modification still needs additional research as well as the investigation of sympatric occurrence of species with completely and incompletely developed laterosensory system.

Additional research on *Phenacogaster* can concentrate on taxa that have not been sampled and additional gene sampling. The presence of two distinct lineages of *P.franciscoensis*, one in the Rio São Francisco and another in the Rio Parnaíba is under investigation. Additional undescribed species are also expected for the genus as we increase taxon sampling in research projects. Finally, further research is required to understand the historical biogeographic processes that contributed to the disjunct distribution of the *Phenacogaster* species across the Brazilian Shield.

## Supplementary Material

XML Treatment for Phenacogaster lucenae

## Supplementary materials

Supplementary material 1 NJ tree of species of *Phenacogaster* Data type phylogenetic Explanation note NJ tree of species of *Phenacogaster*, based on the COI gene. Numbers on branches represent bootstrap support (> 50%) based on 1000 bootstrap pseudoreplicates. Note the position of the new species *Phenacogasterlucenae*. https://binary.pensoft.net/file/855126This dataset is made available under the Open Database License (http://opendatacommons.org/licenses/odbl/1.0/). The Open Database License (ODbL) is a license agreement intended to allow users to freely share, modify, and use this Dataset while maintaining this same freedom for others, provided that the original source and author(s) are credited.Camila S. Souza, George M. T. Mattox, George Vita, Luz E. Ochoa, Bruno F. Melo, Claudio Oliveira

Supplementary material 2 Best-score results of Assemble Species by Automatic Partitioning (ASAP) delimitation of species of *Phenacogaster* Data type analysis https://binary.pensoft.net/file/855127This dataset is made available under the Open Database License (http://opendatacommons.org/licenses/odbl/1.0/). The Open Database License (ODbL) is a license agreement intended to allow users to freely share, modify, and use this Dataset while maintaining this same freedom for others, provided that the original source and author(s) are credited.Camila S. Souza, George M. T. Mattox, George Vita, Luz E. Ochoa, Bruno F. Melo, Claudio Oliveira

Supplementary material 3 Poisson Tree Processes (PTP) delimitation of species of *Phenacogaster* Data type analysis https://binary.pensoft.net/file/855128This dataset is made available under the Open Database License (http://opendatacommons.org/licenses/odbl/1.0/). The Open Database License (ODbL) is a license agreement intended to allow users to freely share, modify, and use this Dataset while maintaining this same freedom for others, provided that the original source and author(s) are credited.Camila S. Souza, George M. T. Mattox, George Vita, Luz E. Ochoa, Bruno F. Melo, Claudio Oliveira

Supplementary material 4 List of the specimens included in the species delimitation analyses Data type table (docx file) https://binary.pensoft.net/file/855129This dataset is made available under the Open Database License (http://opendatacommons.org/licenses/odbl/1.0/). The Open Database License (ODbL) is a license agreement intended to allow users to freely share, modify, and use this Dataset while maintaining this same freedom for others, provided that the original source and author(s) are credited.Camila S. Souza, George M. T. Mattox, George Vita, Luz E. Ochoa, Bruno F. Melo, Claudio Oliveira
