# Supplementary figures and images for: ﻿Molecular species delimitation and description of a new species of Phenacogaster (Teleostei, Characidae) from the southern Amazon basin
Source: Zookeys. 2023 May 26;1164:1–21. doi: 10.3897/zookeys.1164.102436 (PMC10239021; doi:10.3897/zookeys.1164.102436)

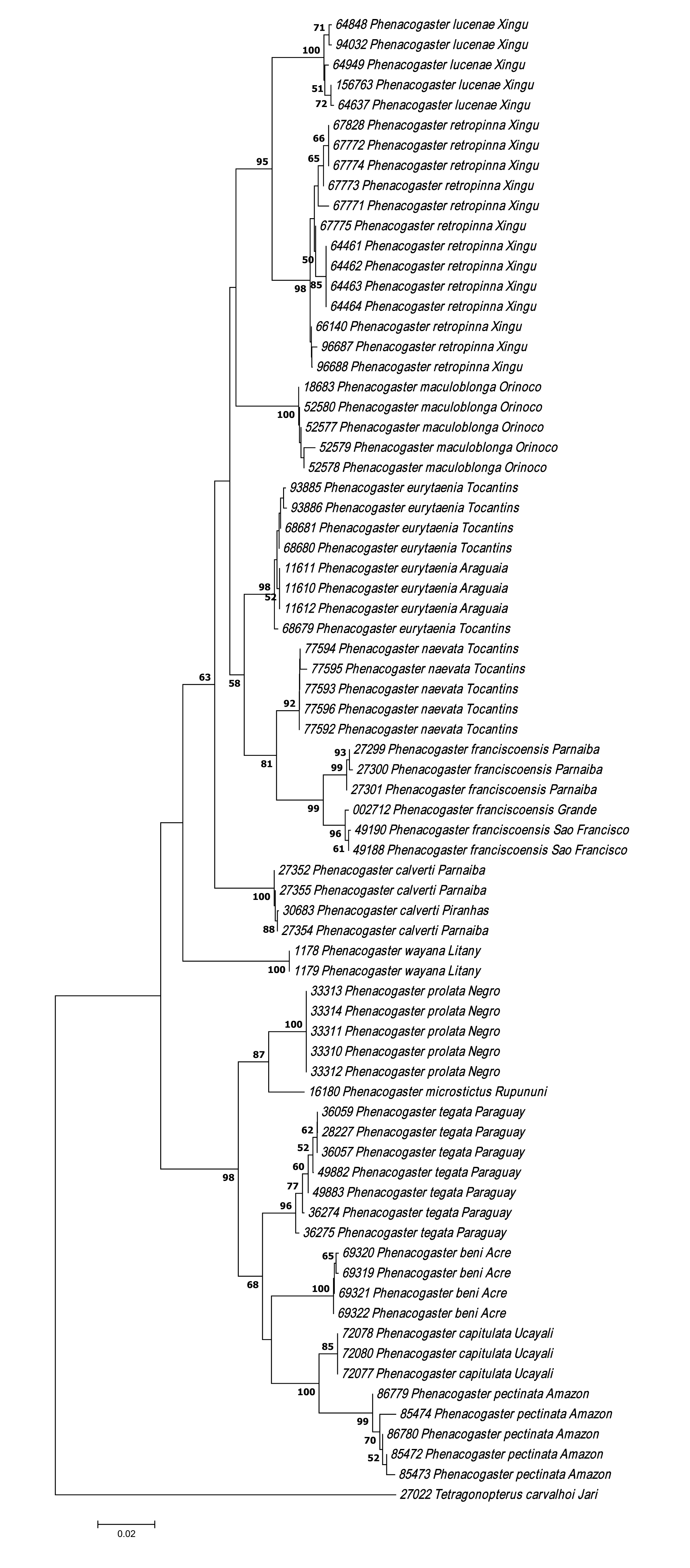

Supplement: Supplementary material 1 — NJ tree of species of Phenacogaster [file zookeys-1164-001_article-102436__-s001.tif]

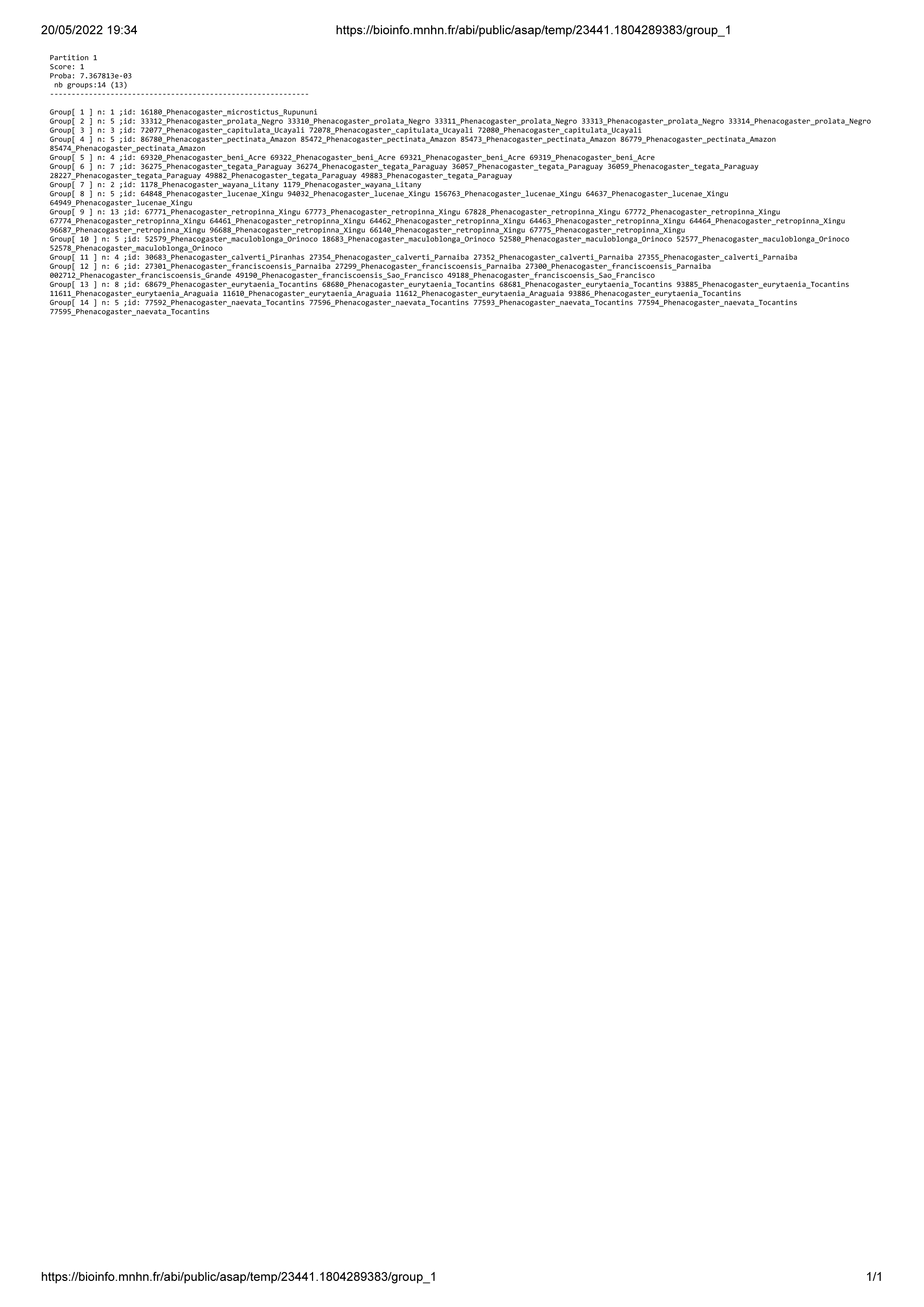

Supplement: Supplementary material 2 — Best-score results of Assemble Species by Automatic Partitioning (ASAP) delimitation of species of Phenacogaster [file zookeys-1164-001_article-102436__-s002.tif]

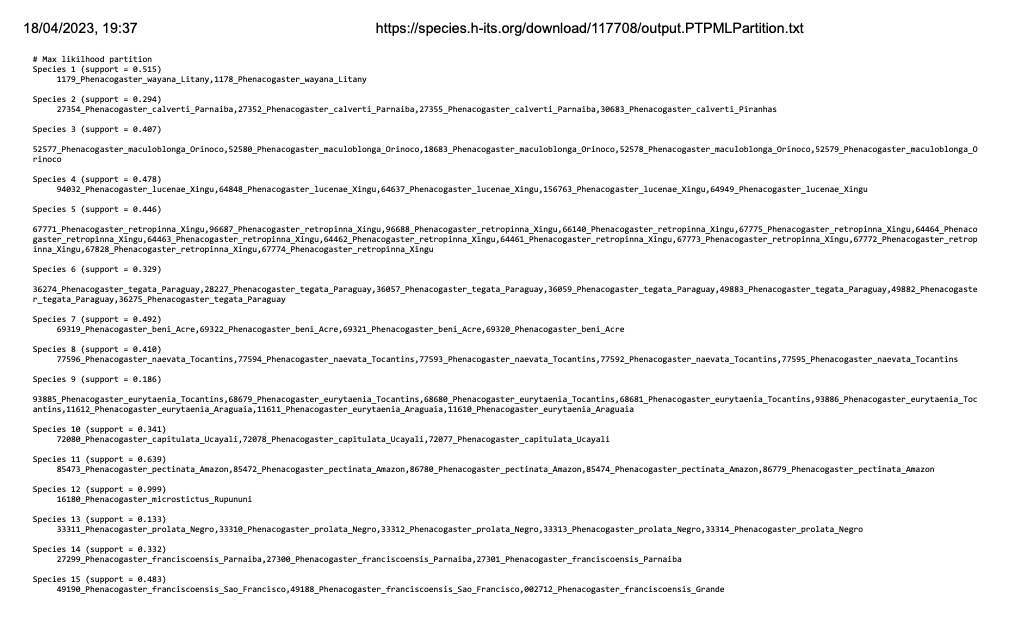

Supplement: Supplementary material 3 — Poisson Tree Processes (PTP) delimitation of species of Phenacogaster [file zookeys-1164-001_article-102436__-s003.png]
