# Supplementary material for: ﻿Molecular species delimitation and description of a new species of Phenacogaster (Teleostei, Characidae) from the southern Amazon basin
Source: Zookeys. 2023 May 26;1164:1–21. doi: 10.3897/zookeys.1164.102436 (PMC10239021; doi:10.3897/zookeys.1164.102436)
Supplement: Supplementary material 4 — List of the specimens included in the species delimitation analyses [file zookeys-1164-001_article-102436__-s004.docx]

Supplementary Table 1. List of the specimens included in the species delimitation analyses.

| **Museum number** | **Voucher** | **Species** | **Drainage** | **Country** | **Coordinates** | **GenBank number** |
| --- | --- | --- | --- | --- | --- | --- |
| LBP 19200 | 77592, 77593, 77594, 77595, 77596 | *P. naevata* | Tocantins River | Brazil | 11°03'14.3''S 48°34'22.0"W | OQ445479, OQ445481, OQ445482, OQ445483, OQ445480 |
| LBP 10487 | 49188, 49190 | *P. franciscoensis* | São Francisco River | Brazil | 17°14'32.3'S 46°28'03.8''W | OQ445470, OQ445469 |
| MZUSP 114504 | 002712 | *P. franciscoensis* | Grande River | Brazil | 12°17'57.7"S 45°00'56.8"W | OQ445468 |
| LBP 5582 | 27299, 27300, 27301 | *P. franciscoensis* | Parnaíba River | Brazil | 09°09'51''S 45°51'15''W | OQ445466, OQ445467, OQ445465 |
| LBP 1507 | 11610, 11611, 11612 | *P. eurytaenia* | Araguaia River | Brazil | 15°52'40.4'' 52°18'15.5''W | OQ445476, OQ445475, OQ445477 |
| LBP 17173 | 68679, 68680, 68681 | *P. eurytaenia* | Tocantins River | Brazil | 14°58'37.4''S 48°40'42.1''W | OQ445471, OQ445472, OQ445473 |
| LBP 25604 | 93885, 93886 | *P. eurytaenia* | Tocantins River | Brazil | 12°37'27.9"S 48°02'43.5"W | OQ445474, OQ445478 |
| LBP 15966 | 66140 | *P. retropinna* | Xingu River | Brazil | 13°29'41.8"S 53°04'57.7"W | OQ445456 |
| LBP 15676 | 64461, 64462, 64463, 64464 | *P. retropinna* | Xingu River | Brazil | 13°09'13.6"S 51°55'18.7"W | OQ445449, OQ445450, OQ445451, OQ445452 |
| LBP 25926 | 96687, 96688 | *P. retropinna* | Xingu River | Brazil | 13°50'50.8''S 53°15'40.2''W | OQ445453, OQ445455 |
| LBP 16691 | 67828 | *P. retropinna* | Xingu River | Brazil | 03°24'19.8''S 52°05'48.0''W | OQ445446 |
| LBP 16670 | 67771, 67772, 67773, 67774, 67775 | *P. retropinna* | Xingu River | Brazil | 03°30'14.3''S 52°02'19.9''W | OQ445444, OQ445447, OQ445445, OQ445448, OQ445454 |
| LBP 18683 | 52577, 52578, 52579, 52580,18683 | *P. maculoblonga* | Orinoco River | Colômbia | 3°16'17.4"N 73°36'47.0"W | OQ445460, OQ445461, OQ445457, OQ445459, OQ445458 |
| LBP 15734 | 64637 | *P. lucenae* | Xingu River | Brazil | 12°53'04.3"S 52°02'00.3"W | OQ445443 |
| LBP 25217 | 94032 | *P. lucenae* | Xingu River | Brazil | 08°39'06.9''S 55°02'09.1''W | OQ445440 |
| LBP 15807 | 64848 | *P. lucenae* | Xingu River | Brazil | 12°33'20.5"S 52°16'16.1"W | OQ445439 |
| LBP 15835 | 64949 | *P. lucenae* | Xingu River | Brazil | 12°31'55.7"S 52°20'29.8"W | OQ445441 |
| LBP 15676 | 156763 | *P. lucenae* | Xingu River | Brazil | 13°09'13.6"S 51°55'18.7"W | OQ445442 |
| MCP 30683 | 30683 | *P. calverti* | Piranhas River | Brazil | 6°43'32.0"S 37°47'50.0"W | FJ749077 |
| LBP 5612 | 27352, 27354, 27355 | *P. calverti* | Parnaiba River | Brazil | 7°30'55.0"S 46°04'53.0"W | OQ445463, OQ445462, OQ445464 |
| MHNG 2759.079 | GBOL1178, GBOL1179 | *P. wayana* | Litany River | French Guiana | 2°56'16.7"N 54°10'20.6"W | MZ051714, MZ051920 |
| LBP 6931 | 33310, 33311, 33312, 33313, 33314 | *P. prolata* | Negro River | Brazil | 0°04'66.5"N 66°48'54.6"W | OQ445416, OQ445417, OQ445415, OQ445418, OQ445419 |
| ROM uncat | 16180 | *P. microstictus* | Essequibo River | Guyana | 2°09'33.5"N 59°17'33.5"W | OQ445414 |
| LBP 22680 | 85472, 85473, 85474 | *P. pectinata* | Amazon River | Brazil | 4°12'02.7"S 69°55'35.3"W | OQ445424, OQ445425, OQ445427 |
| LBP 22418 | 86779, 86780 | *P. pectinata* | Amazon River | Colômbia | 4°07'33.8"S 70°00'28.9"W | OQ445426, OQ445423 |
| LBP 17802 | 72077, 72078, 72080 | *P. capitulata* | Ucayali River | Peru | 8°35'44.2"S 74°48'04.3"W | OQ445420, OQ445421, OQ445422 |
| LBP 5795 | 28227 | *P. tegata* | Aricá-Mirim River | Brazil | 15°44'03.6"S 55°52'48.7"W | OQ445436 |
| LBP 10785 | 49882, 49883 | *P. tegata* | Paraguay River | Brazil | 18°25'24.4"S 54°50'05.9"W | OQ445438, OQ445437 |
| LBP 7606 | 36274, 36275 | *P. tegata* | Paraguay River | Brazil | 16°11'39.5"S 55°48'25.1"W | OQ445433, OQ445432 |
| LBP 7641 | 36057, 36059 | *P. tegata* | Paraguay River | Brazil | 15°46'03.8"S 55°30'44.5"W | OQ445434, OQ445435 |
| LBP 16865 | 69319, 69320, 69321, 69322 | *P. beni* | Acre River | Brazil | 10°04'44.3"S 67°32'33.9"W | OQ445431, OQ445428, OQ445430, OQ445429 |
| LBP 5376 | 27022 | *T. carvalhoi* | Jari River | Brazil | 0°33'51.0"S 52°34'45.0"W | HM070393.1 |
